# Annual Research Review: The persistent and pervasive impact of being bullied in childhood and adolescence: implications for policy and practice

**DOI:** 10.1111/jcpp.12841

**Published:** 2017-11-14

**Authors:** Louise Arseneault

**Affiliations:** Institute of Psychiatry, Psychology and Neuroscience, King’s College of London, London, UK

**Keywords:** Bullying victimisation, mental health, physical health, socioeconomic outcomes, development, children, adolescents, life course

## Abstract

**Background:**

We have known for some time that being bullied was associated with children’s and adolescents’ adjustment difficulties and well-being. In recent years, we have come to recognise that the impact of childhood bullying victimisation on the development of mental health problems is more complex. This paper aims to review the evidence for an independent contribution of childhood bullying victimisation to the development of poor outcomes throughout the life span, including mental, physical and socioeconomic outcomes, and discuss the implications for policy and practice.

**Findings:**

Existing research indicates that (a) being bullied in childhood is associated with distress and symptoms of mental health problems. This large body of evidence supports actions aimed at reducing the occurrence of bullying behaviours; (b) the consequences of childhood bullying victimisation can persist up to midlife and, in addition to mental health, can impact physical and socioeconomic outcomes. These new findings indicate that interventions should also focus on supporting victims of bullying and helping them build resilience; (c) research has identified some factors that predispose children to be targeted by bullying behaviours. These studies suggest that public health interventions could aim at preventing children from becoming the target of bullying behaviours from an early age.

**Conclusions:**

It is a truism to emphasise that further work is needed to understand why and how young people’s aspirations are often cut short by this all too common adverse social experience. In parallel, we must develop effective strategies to tackle this form of abuse and its consequences for the victims. Addressing bullying in childhood could not only reduce children’s and adolescents’ mental health symptoms but also prevent psychiatric and socioeconomic difficulties up to adulthood and reduce considerable costs for society.

## Introduction

There is little doubt today that being bullied is an adverse and stressful experience that casts a shadow on children’s and adolescents’ well-being and development. But this has not always been the view. After several years of general scepticism about the true impact of bullying victimisation, it is only recently that researchers, mental health professionals and policy makers have started to pay attention to the potentially harmful consequences of being bullied in early life. This change in perception is reflected in different ways. First, the number of publications on the topic of bullying has grown exponentially since the early 1990s (see Olweus, 2013). This accumulating evidence indicates that young victims of bullying are at risk of showing adjustment problems and even developing severe mental health problems. Second, another important consequence of increasing concerns relating to the impact of childhood bullying victimisation is the development of intervention programmes designed specifically to limit bullying behaviours at schools. The efficiency of those programmes has been reviewed in meta-analytic studies that have reported mixed results (Ttofi & Farrington, 2011). Third, national policies have also responded to society’s greater awareness of bullying. In the United Kingdom, all schools have a legal obligation to have measures in place to prevent and handle forms of bullying among pupils and to inform teachers, pupils and parents about these measures (Department for Education, 2017). In the United States, more than 120 bills related to antibullying policies were adopted between 1999 and 2010 and a total of 49 states have laws in place to tackle bullying behaviours at school (Hatzenbuehler, Schwab-Reese, Ranapurwala, Hertz, & Ramirez, 2015). However, despite joint efforts to reduce bullying and understand its consequences for the victims, this behaviour remains frequent among young people.

This review paper aims to summarise findings on the impact of being bullied from population-based samples with prospective measures of bullying victimisation in childhood or early adolescence. It emphasises longitudinal studies that examined mental health and other outcomes up to adulthood, and considers how these findings may influence policy and practice. It also aims to provide pointers for future research. This review paper does not report on children who bully others or focus on the dyadic relationship between them and their victims. It does not focus on bullying victimisation among specific groups such as children with developmental disorders or disabilities, for example. This paper considers bullying as a global form of abuse and does not distinguish specific types of bullying victimisation. This review paper is timely in light of the emphasis of current policies on youth mental health. It summarises the body of evidence so far on one of the most prevalent risk factors for mental health problems in childhood and adolescence. It also builds upon review papers published recently on the long-term outcomes of being bullied (Brunstein Klomek, Sourander, & Elonheimo, 2015; McDougall & Vaillancourt, 2015; Wolke & Lereya, 2015) and expands by raising important questions for policy and practice: are we doing the right thing? Are we doing enough? This review is also timely as we immerse ourselves in a new digital age which allows harassment and bullying to be more insidious, as summarised by a previous review paper published in this journal (Livingstone & Smith, 2014).

## What is bullying?

Bullying victimisation is the repeated occurrence of abuse between people from the same age group where an imbalance of power makes it difficult for the victims to defend themselves (Olweus, 1993, 2013). Bullying, a form of peer victimisation, can take place between children, between adolescents or between adults. It is not bullying when a parent or a teacher is abusive towards a child. While the terms *peer victimisation* and *bullying* are often used interchangeably, peer victimisation is not equivalent to bullying. For example, it is not bullying when two people of about the same strength quarrel or fight, but it is peer victimisation. An especially important feature of bullying is the power imbalance between those who perpetrate bullying behaviours and their victims. Strength, number or size of those involved can place the victims at a disadvantage. The power imbalance can also be more subjective and difficult to capture, involving factors such as popularity, intelligence or disabilities. It can also be determined by the environment: a child who just joined a new school may be at risk of being bullied by others, as would a child belonging to a minority group. Dan Olweus, the founder of research on bullying, argued that the power imbalance is best determined by the victims themselves (2013). Victims of bullying can also bully other vulnerable youths. ‘Bully/victims’ represent a small but distinct group of children who are involved in bullying both as a perpetrator and as a victim. The distinction between bullying and peer victimisation may appear trivial or pedantic but it is important when investigating the consequences of this form of abuse. By definition, victims of bullying represent a group of individuals who, for various reasons, are less likely to retaliate when confronted with abusive behaviours from their peers. They constitute a heterogeneous and vulnerable group who might be likely to experience adversity, adjustment difficulties or even mental health problems at some point in their lives, despite the experience of bullying. It is therefore reasonable to question whether the sheer act of being bullied truly contributes to poor outcomes among the victims, and if so, how.

Determining the impact of childhood bullying victimisation on children’s and adolescents’ mental health and well-being, as well as reducing the occurrence of bullying behaviours, are important for several reasons. First, bullying is common world-wide among children and adolescents. A survey of children in nearly 40 countries indicated that approximately 13% of 11-year-olds reported being the victims of bullying (World Health Organisation, 2012). Prevalence rates vary greatly across countries, are commonly higher for boys compared to girls, and decline with age. Rates across 11 European countries revealed a similar pattern: 20% of youth from 8 to 18 reported being bullied (Analitis et al., 2009); bullying victimisation was more prevalent among boys and tended to decline with age. In the United Kingdom and in the United States, bullying, including peer and sibling victimisation, is the most prevalent form of abuse across all age groups up to 24 years (Finkelhor, Ormrod, & Turner, 2007a; Radford, Corral, Bradley, & Fisher, 2013). These prevalence rates reflect an increase in bullying awareness which contrasts with early research when bullying was studied almost exclusively in Scandinavian countries (Olweus, 1993). Second, bullying is widespread across different environments. It most commonly takes place in schools, but bullying can also occur in other contexts, including in the neighbourhood or at home between siblings (Wolke & Skew, 2012a). Third, bullying can be persistent across time and across settings (Sourander, Helstelä, Helenius, & Piha, 2000). Chronic victimisation is not infrequent, even despite the transition to secondary school during the early teenage years: of the children who were frequently bullied during primary school in the United Kingdom, 43.1% of boys and 40.1% of girls remained frequently bullied during secondary school (Bowes et al., 2013). These findings are in line with a previous study showing that nearly half of age-11 young victims of bullying (43%) were still victims 3 years later (Scholte, Engels, Overbeek, de Kemp, & Haselager, 2007). Of the children who were not involved in bullying at the first assessment, only 7% became victims later on. Lower stability in bullying victimisation has also been reported (Schäfer, Korn, Brodbeck, Wolke, & Schulz, 2005). These contrasting findings are possibly accounted for by the relatively short reporting periods covered by the assessments. Fourth, bullying can take various forms. It can be verbal such as threatening, taunting, spreading rumours or it can refer to physical actions including pushing and kicking. It can be direct (e.g. verbal and physical behaviours conducted in the context of face-to-face interactions) or indirect (e.g. actions that do not necessarily require the bullies and the victims to be present, like spreading rumours and excluding others). Fifth, bullying has evolved with time. New technologies and social media platforms, easily accessible via mobile phones or the Internet, provide countless opportunities for young people to bully and damage the reputations of their victims, in front of large crowds of witnesses who may exacerbate the abuse. Cyberbullying has been documented as a new and harmful form of bullying, especially among adolescents (Smith et al., 2008).

## Adjustment problems associated with bullying victimisation

As with victims of crimes or assaults, children and adolescents are likely to get upset when targeted by abusive behaviours. Young victims can manifest signs of psychological distress such as being tearful or irritable, losing motivation and experiencing sleep problems. These could be considered as temporary reactions to a stressful event and would normally recede with appropriate support when exposure to bullying behaviours cease. Documented reactions associated with bullying victimisation include being unhappy at school, difficulties in school adjustment and poor school perceptions (Arseneault et al., 2006; Glew, Fan, Katon, & Rivara, 2008; Juvonen, Graham, & Schuster, 2003; Nansel, Craig, Overpeck, Saluja, & Ruan, 2004), facing social problems such as being isolated and feeling lonely (Juvonen et al., 2003; Kaltiala-Heino, Rimpelä, Rantanen, & Rimpelä, 2000; Nansel et al., 2001, 2004; Scholte et al., 2007; Veenstra et al., 2005), and academic difficulties (Bowes et al., 2013; Glew et al., 2008).

Victims of bullying can also manifest symptoms of psychological distress commonly associated with psychopathology. Studies have found that bullied youth showed an increased risk of self-harm and suicidal ideation (Barker, Arseneault, Brendgen, Fontaine, & Maughan, 2008; Geoffroy et al., 2016; Lereya, Winsper et al., 2013; Sibold, Edwards, Murray-Close, & Hudziak, 2015; Turner, Exum, Brame, & Holt, 2013; Winsper, Leraya, Zanarini, & Wolke, 2012), and especially among those victims who experienced mental health problems, felt rejected at home or were maltreated by an adult, had parents with emotional problems, or had a family history of attempted or completed suicide (Fisher et al., 2012; Herba et al., 2008). Severe symptoms of psychological distress are thus concentrated among bullied youth who show a range of risk factors for mental health problems. While common signs of psychological distress among victims of bullying may not require clinical interventions, more severe manifestations including self-harm and suicidal ideation signal a profound impact among some of those targeted by those who bully others. Such symptoms necessitate prompt and adequate interventions by mental health professionals. These also point towards a severe impact of bullying victimisation on mental health problems in childhood and adolescence.

## Contribution of bullying victimisation to the development of mental health problems in childhood and adolescence

Longitudinal study designs are instrumental for establishing the extent to which being the victim of bullying is a contributing risk factor to the development of mental health problems. Establishing temporal priority – what come first, bullying victimisation or poor mental health – is an essential first step. Indeed, one important alternative hypothesis that must be ruled out is that early mental health symptoms account for both an increased risk for being targeted by bullying behaviours and also for later psychopathology. Findings so far have shown that over and above early signs of poor mental health prior to bullying victimisation, being bullied in childhood or in adolescence is associated with new symptoms/diagnoses of mental health problems, and especially with later symptoms of anxiety and depression (Arseneault et al., 2006; Bowes, Joinson, Wolke, & Lewis, 2015; Kim, Leventhal, Koh, Hubbard, & Boyce, 2006; Stapinski et al., 2014; Zwierzynska, Wolke, & Leraya, 2013). These studies are robust not only because they controlled for symptoms prior to being bullied but they also controlled for a range of other potential confounders, including gender, parental socioeconomic status and low IQ. Bullying victimisation has also been associated with symptoms of rare mental health problems in adolescence such as psychotic experiences: bullied youth, and especially those who were frequently or severely bullied, have an increased risk for reporting psychotic experiences in adolescence (Arseneault et al., 2011; Cunningham, Hoy, & Shannon, 2016 for a review; Kelleher et al., 2013; Mackie, Castellanos-Ryan, & Conrod, 2011; Schreier et al., 2009). One exception is a study that reported no association between bullying victimisation in adolescence and psychotic experiences after controlling for childhood behavioural problems and other forms of victimisation (Boden, van Stockum, Horwood, & Fergusson, 2016). This finding is possibly explained by the relatively small number of youth who were exposed to a ‘high level’ of bullying in this sample.

The extent to which being the victim of bullying contributes to the development of mental health problems in childhood and adolescence has critical implications for prevention and intervention efforts. Although these strategies are important to safeguard the human rights of children, reducing bullying behaviour could be an expensive and ineffective way of decreasing children’s early symptoms of poor mental health if being bullied is spuriously associated with poor outcomes. Strong and robust tests supporting the assumption that being bullied in childhood can actually contribute to mental health problems remain sparse. One reason for this is the limits of observational studies most commonly used to examine the outcomes associated with being bullied in childhood and adolescence. Randomised controlled trials would allow proper testing for a possible causal role of bullying victimisation, but randomly assigning children to bullied and nonbullied conditions is not an option for obvious ethical reasons. Researchers therefore have to resort to using alternative study designs and statistical methods (Jaffee, Strait, & Odgers, 2012; Rutter, Pickles, Murray, & Eaves, 2001) to strengthen the evidence clarifying the role of bullying victimisation for the development of mental health problems. The discordant monozygotic (MZ) twin design offers a rigorous control for confounders by contrasting genetically identical individuals drawn from the same family environment but who are exposed to distinct experiences (Vitaro, Brendgen, & Arseneault, 2009). Because many early family experiences are necessarily the same within pairs of twins who grow up together, shared environmental factors such as poverty, domestic violence or maternal depression cannot account for the differences in the outcome variables. Furthermore, because MZ twins are genetically identical, variation in outcomes cannot be the result of genetic variations between the two twins either. Therefore, the discordant MZ twin design can be used to test whether being bullied in childhood has an environmentally mediated impact on the development of mental health symptoms at a young age, over and above shared environmental and genetic confounds. When applied to longitudinal data, the discordant MZ twin design is a powerful methodological tool for investigating the pathway from bullying victimisation to children’s developmental outcomes.

Three longitudinal studies have used the discordant MZ twin design to test the robustness of the impact of being bullied in childhood on mental health outcomes. A first study from the Environmental Risk (E-Risk) Longitudinal Twin Study (Moffitt, 2002) showed that MZ twins who had been bullied by the age of 7 had more emotional problems at age 10 years compared to their cotwins who had not been bullied (Arseneault et al., 2008). This difference remained significant even after controlling for emotional problems assessed when the twins were 5 years of age, prior to being bullied. A second study from the Twins Early Development Study (TEDS; Trouton, Spinath, & Plomin, 2002) found similar findings using a measure of peer victimisation in early adolescence with a larger sample of twins: MZ twin differences in peer victimisation were associated with differences in anxiety over the course of 2 years, even after controlling for prior anxiety, but became nonsignificant over 5 years (Singham et al., 2017). Differences remained significant, however, for measures of paranoid thoughts and cognitive disorganisation (without control for prior measures). These findings may be taken to suggest that the contribution of bullying victimisation to mental health problems is not long-lasting. However, the Virginia Twin Study of Adolescent Behavioral Development (Eaves et al., 1997) indicated otherwise, and extended others’ findings by examining mental health outcomes both in childhood and in young adulthood. Results revealed that compared with their nonbullied cotwins, bullied MZ twins were nearly twice as likely to have social anxiety and separation anxiety in childhood and three times more likely to report suicidal ideation in young adulthood (Silberg et al., 2016). Psychiatric disturbances prior to being bullied did not differ between the bullied and nonbullied twins in this sample and therefore, could not account for differences in outcomes. These three studies robustly demonstrate that bullying victimisation contributes to later mental health outcomes: overall, associations were not explained by prior symptoms or difficulties, and the associations survived strict controls for confounders, including both family background and genetic factors. This evidence suggests that if we eliminate bullying behaviours, we should be successful at reducing mental health problems in youths.

Despite these strong findings, not all bullied children end up developing mental health problems. Studies testing the modifying effect of variables on outcomes associated with bullying victimisation are also important. First, this research may help disentangle and characterise subgroups of youth who are most likely to develop problems as a consequence of being bullied. There are a few examples of such studies focusing on biological factors. One study showed that variation in the serotonin transporter (5-HTTLPR) gene, involved in mood regulation and depression, moderates children’s emotional problems in response to bullying victimisation: frequently bullied children with the SS genotype were at greater risk for developing emotional problems than were children with the SL or LL genotypes (Sugden et al., 2010). Another study indicated that peer victimisation predicted symptoms of depression 1 year later specifically among participants who showed high levels of anticipatory salivary cortisol response (Rudolph, Troop-Gordon, & Granger, 2011). This heightened anticipatory cortisol response protected participants from depressive symptoms when they were exposed to low levels of peer victimisation.

Second, studies of social factors can help identify targets for interventions aimed at reducing symptoms of mental health problems. One study demonstrated that most bullied young adolescents do not engage in self-harming behaviours, but those who did were more likely to have a family member who had attempted/completed suicide, compared to those who did not self-harm (Fisher et al., 2012). They were also more likely to have been physically maltreated by an adult and to present with conduct disorder, borderline personality characteristics, depression and psychotic symptoms. Another study reported that while self-blaming was not associated with a general measure of peer victimisation, children who showed an inclination to blame themselves also showed higher levels of emotional problems if victimised by their peers (Perren, Ettekal, & Ladd, 2013). A further study showed that bullied children who had highly supportive families had fewer emotional and behavioural problems over time compared to those from less supportive families (Bowes, Maughan, Caspi, Moffitt, & Arseneault, 2010). Although maternal warmth, sibling warmth and a positive atmosphere at home were associated with positive adjustment for both bullied and nonbullied children, the effects of these protective family factors were significantly stronger for bullied children compared to those who had not been bullied. Findings from these last two studies have especially important implications for clinical efforts: interventions focusing on negative cognitions and involving families may have greater chances of tackling symptoms of mental health problems among bullied children.

The evidence reviewed thus far indicates that being bullied in childhood is not only associated with signs of psychological distress but also with symptoms of mental health problems in childhood and adolescence. These findings support actions to stop bullying behaviours in order to reduce suffering in youth and prevent the development of mental health problems. Such actions are already in place.

## The persistent effect of childhood bullying victimisation on mental health problems

To date, relatively little is known about the longterm impact of bullying, as only a few longitudinal studies with prospective measures of bullying victimisation in childhood have followed participants into adult life. ‘Long-term’ is characterised here not only by the age of the participants when outcomes were assessed, but also by the time lag between exposure to bullying victimisation and mental health problems. So far, four longitudinal cohorts have documented the adult outcomes of childhood bullying victimisation, at least 10 years apart, with adequate consideration for childhood mental health problems and other confounders. The Epidemiologic Multicenter Child Psychiatric Study is a prospective nationwide birth cohort study from Finland (Almqvist et al., 1999). Information on bullying victimisation was collected from parents, teachers and children themselves in 1989, when the participants were aged 8 years. Findings from this cohort have indicated that girls who were frequent victims of childhood bullying had increased rates of suicide attempts and completed suicides up to age 25 (Brunstein Klomek et al., 2009). Male participants who had been victims of bullying had higher rates of anxiety disorders between ages 18 and 23 years (Sourander, Jensen, Rönning, Niemelä et al., 2007), and increased risk of heavy smoking (Niemelä et al., 2011). Most data on young adult outcomes in these studies were gathered from military call-up, national psychiatric and hospital discharge registers, and thus may underestimate distress, especially among females and victims who did not seek treatment.

This limitation was addressed in an accelerated population-based study with outcome measures collected during research-based assessments, the Great Smoky Mountain Study from North Carolina in the United States (Costello et al., 1996). Information on bullying victimisation was collected on multiple occasions from caregivers and children themselves when the participants were between the ages of 9 and 16. Compared to those who had not been bullied in childhood, victims of bullying, and especially bully/victims, had increased rates of psychiatric disorders including agoraphobia, depression, anxiety and panic disorders in their early to mid 20s, up to 14 years after exposure (Copeland, Wolke, Angold, & Costello, 2013). Participants who had been bullied in childhood also had high rates of suicidality, but not of antisocial personality or substance use disorders.

The long-term impact of childhood bullying victimisation was further investigated in National Child Development Study (NCDS), or the 1958 British Cohort Study, a 50-year prospective followup of a UK birth cohort (Power & Elliott, 2006). Information on bullying victimisation was collected from parents when participants were aged 7 and 11, in 1965 and 1969. Analyses were undertaken first to ensure that bullying victimisation assessed in the mid-1960s referred to the same concept as bullying today: reassuringly, findings indicated that as shown by other contemporaneous studies, bullying victimisation was associated with known childhood correlates including low parental socioeconomic status, low IQ, as well as emotional and behavioural problems. Supporting the findings from the two other cohorts, but extending them through the inclusion of outcomes at midlife, the NCDS study showed that victims of bullying in childhood reported high levels of psychological distress not only at age 23 but also, and most importantly, at age 50, nearly 40 years after exposure (Takizawa, Maughan, & Arseneault, 2014). Participants who had been victims of bullying in childhood had higher prevalence of psychiatric disorders in midlife, including depression and anxiety, compared to participants who had not been bullied. The effects were small but similar to those associated with other adverse childhood exposures measured in this cohort study such as placement in care or exposure to multiple adversities within the family. Strikingly similar to findings from the United States, participants in NCDS who had been bullied in childhood had increased rates of suicidality, but not of alcohol dependence.

The fourth birth cohort study partially corroborates the pattern of findings observed so far. The Christchurch Child Development Study is a longitudinal examination of 1265 individuals born in Christchurch New Zealand, in 1977 (Fergusson, Horwood, Shannon, & Lawton, 1989). Data on bullying victimisation were collected when participants were aged 13, 14 and 15 by asking their parents whether they experienced problems at school including ‘being teased, bullied by other children’. Participants reported on mental health outcomes at ages of 16–21, 21–25 and 25–30. Bullying victimisation and outcome measures were pooled across age periods and may blur the longterm impact investigated here. Findings indicated that victims of bullying had an increased risk for anxiety disorder in later years (Gibb, Horwood, & Fergusson, 2011). Further tests with other mental health outcomes including depression, and suicidal thoughts and attempts did not survive controls for confounders. The small number of participants who had been bullied (*N* = 30) and the reporting period covering mostly the adolescent years, may explain the dissimilarity in the conclusions.

The findings reported here are based on observational data and thus do not allow causal inferences. The consistency of the findings across the four cohorts is, however, compelling. These studies (a) used prospective measures of bullying victimisation in childhood and later outcomes in adulthood; (b) controlled for mental health problems in childhood, indicating that bullying victimisation contributes either to the onset or worsening of mental health problems in later years; (c) accounted for a range of confounders that might also explain poor later outcomes in young victims of bullying, including childhood IQ, parental SES, other forms of adversities and gender; and (d) are representative of the populations of four different countries. Conclusions from these studies cannot be ignored. Taken together, these findings suggest that the impact of bullying on the young victims may persist once the bullying has long stopped. Tackling bullying behaviours may not only reduce children’s and adolescents’ mental health symptoms and adjustment difficulties, but also prevent psychiatric problems in adulthood. Furthermore, if symptoms persist beyond the childhood and adolescent periods, this indicates that support to young victims, even after the bullying has stopped, is necessary to reduce the long-term burden of mental health difficulties among young victims of bullying.

## Beyond mental health problems: physical health, criminal and socioeconomic outcomes

The long-term impact of bullying victimisation explored by the four longitudinal cohorts described above was not limited to mental health problems. Focusing on outcomes in the adult years opens up the possibility of examining a range of life domains more difficult to study in childhood or adolescence. These are physical health, criminal and socioeconomic domains.

Examining physical health outcomes associated with bullying victimisation among children and adolescents is challenging as most chronic diseases are relatively rare at this young age and risk indicators may still be latent. With higher prevalence rates of diseases, the midlife period offers the possibility of robustly exploring these long-term outcomes. Findings from NCDS indicated that being bullied in childhood was associated with self-ratings of poor general health at age 50 (Takizawa et al., 2014) and this finding provided the basis for investigating physical health in greater depth and detail. A follow-up study indicated that men and women who had experienced bullying victimisation in childhood showed higher inflammation levels than nonbullied peers, while women who had been bullied were more likely to be obese decades later (Takizawa, Danese, Maughan, & Arseneault, 2015). Findings were consistent across two different measures of inflammation (C-reactive protein (CRP) and fibrinogen) and two different measures of adiposity (BMI and waist-hip ratio). Findings were independent of the effects of correlated childhood risks (e.g. parental social class, participants’ BMI and psychopathology in childhood), and of key adult risk factors targeted by current preventive interventions for obesity or cardiovascular disease (e.g. not only smoking, diet and exercise but also adult social class). These markers of poor physical health among victims of bullying were also observed at a younger age in two studies. First, participants from the Great Smoky Mountain Study who were bullied in childhood showed a greater increase in low-grade systemic inflammation (as indexed with CRP levels) from childhood to adulthood (ages 19 and 21), compared to those participants who had not been bullied (Copeland et al., 2014). Second, children who were chronically bullied from primary to secondary schools were nearly twice as likely to be overweight at age 18 than nonbullied children, independently of co-occurring maltreatment, child socioeconomic status, food insecurity, mental health, cognition, pubertal development, childhood weight, and genetic and fetal liability (Baldwin et al., 2016).

Criminal outcomes have been associated with bullying victimisation, but more specifically with bully/victims. Boys who both were frequently bullied by others and who also bullied others in childhood had an increased risk for repeated offending when they were aged 16–20 years according to the Finnish National Police Register data (Sourander, Jensen, Rönning, Elonheimo et al., 2007). This risk was concentrated among those who had psychiatric problems, indicating that the likelihood of committing criminal behaviours in later life among victims of bullying was limited to a minority who also bullied others and who had mental health problems. A follow-up study confirmed the associations between bullying perpetration and criminal offenses between 23 and 26 years among men, but no increased risk was found for those who were solely victims of bullying (Sourander et al., 2011). Although bully/victims did not have an increased risk of meeting diagnostic criteria for antisocial personality disorders in their mid-20s (Copeland et al., 2013), they were more likely to have received felony charges according to courts records (Wolke, Copeland, Angold, & Costello, 2013). Bully/victims were not examined in the Christchurch cohort, but findings indicated that victims of bullying had an increased risk of self-reported property offending (Gibb et al., 2011). This finding is at odds with those of the Finnish and the American cohorts which both found that individuals who were solely victims of bullying were not at increased risk of committing risky or illegal behaviours in late adolescence or during their adult years.

The impact of bullying victimisation has further been found to extend to economic hardship, social relationships and perceived quality of life in the adult years. Individuals who had been bullied in childhood had difficulties keeping jobs in young adulthood (Wolke et al., 2013) and were more likely to be unemployed at midlife (Takizawa et al., 2014). These difficulties remaining active on the job market are not surprising in light of victims’ academic problems. Indeed, those who were frequently bullied had lower educational levels at midlife (Brown & Taylor, 2008; Takizawa et al., 2014). Young victims of bullying also saw their social relationships affected in later years: individuals who had been bullied in childhood had problems making or keeping friends in their mid-20s, and had poor relationships with their parents (Wolke et al., 2013). They had an increased risk of living without a spouse or partner at age 50, they were less likely to have met up with friends in the recent past, and were less likely to have access to social support if they were sick (Takizawa et al., 2014). Finally, bullying victimisation also affected adult well-being: being bullied was associated with lower perceived quality of life at age 50 and lower satisfaction with life so far. Those who had been frequently bullied also anticipated less life satisfaction in the years to come (Takizawa et al., 2014).

The consistency of findings with regard to poor physical and socioeconomic outcomes observed among victims of bullying, across ages and across cohorts, is again striking. It is important to note, however, that poor long-term outcomes were observed especially for those who were frequently or chronically bullied in childhood, and in the case of criminal outcomes, more often among those who were bully/victims. Taken together, these findings suggest that childhood bullying victimisation is not only associated with individual suffering but could also be linked to considerable costs for society given its pervasive impact on physical, criminal and socioeconomic outcomes. Some studies have already pointed out the consequences of childhood bullying victimisation on the health care system. The Finnish birth cohort showed that participants who were frequently bullied in childhood were more likely to have received psychiatric hospital treatment and used psychiatric medications at age 24, over and above psychopathology prior to bullying (Sourander et al., 2009). These effects on service use were shown to be persistent: being frequently bullied in childhood was associated with treatment for psychiatric disorders at age 29, over and above family factors and childhood psychiatric symptoms (Sourander, Gyllenberg et al., 2016). Using data from NCDS, a study reported that compared to participants who were not bullied in childhood, those who were frequently bullied were more likely to use mental health services in childhood, adolescence and also in midlife (Evans-Lacko et al., 2016). This disparity in service use associated with childhood bullying victimisation was explained both by new use of mental health services up to age 33 by a subgroup of participants, and also by persistent use up to midlife.

Similar to children and adolescents who suffered from maltreatment, young victims of bullying may need support to overcome their difficulties facing this stressful situation. Appropriate interventions may be as simple as schools and families acknowledging the impact of being bullied to prevent normal reactions of distress from developing into mental health problems (Leff & Waasdorp, 2013). Studies have highlighted the important role of families in building resilience among bullied victims (Bowes et al., 2009, 2013; for a review see Lereya, Samara, & Wolke, 2013). Increasing families and school awareness of the damaging impact associated with bullying victimisation is essential to detect early signs of distress among young victims of bullying. More targeted interventions by mental health professionals may also be required in instances where symptoms of mental health problems have emerged. These symptoms should not be overlooked even if the bullying behaviours have stopped. Interventions in the adult years may also help with reversing the harmful impact of bullying when the victims enter adulthood. However, no studies have yet tested this hypothesis.

## Mechanisms accounting for poor outcomes among young victims of bullying: further targets for building resilience

The evidence supporting the persistent impact of bullying victimisation on poor outcomes up to adulthood is intriguing. However, the developmental processes that translate childhood bullying victimisation into poor outcomes up to adulthood remain unclear. How can abusive behaviours perpetrated by other pupils and classmates leave marks observable well into adult life? We need a better understanding of these interactive processes to identify specific targets for intervention programmes aimed at reducing the harmful outcomes of being bullied and building resilience among young victims.

Two possible processes that have been examined refer to hypotheses derived from theories of the biological embedding of stress (Danese & McEwen, 2012). One such process relates to variation in the hypothalamic–pituitary–adrenal (HPA) axis activity, commonly associated with the neurobiology of stress. A study from the E-Risk cohort using a group of MZ twins discordant on bullying victimisation showed that bullying victimisation in childhood was associated with a blunted salivary cortisol response (Ouellet-Morin, Danese et al., 2011), which in turn, was associated with problems with social interactions and aggressive behaviours among children who were victims of bullying or physical maltreatment (Ouellet-Morin, Odgers et al., 2011). These findings are in line with other studies showing an association between bullying victimisation and daily hyposecretion of cortisol among girls (Vaillancourt et al., 2008) and also among adolescents following laboratoryinduced stressful situation (Calhoun et al., 2014). But what processes might activate this reduction in cortisol level after children have experienced violence repeatedly over time? Using the same group of discordant MZ twins from the E-Risk cohort, a further study showed that the bullied twins had higher methylation levels on *5-HTTLPR* compared to their nonbullied cotwins (Ouellet-Morin et al., 2013). In addition, findings from this study showed that higher levels of methylation were associated with lower levels of cortisol response. Effects of this kind may serve as an interface between childhood bullying victimisation and later vulnerability to stress and psychopathology. Interventions focussed on teaching coping skills for dealing with stressful situations and managing stress reactions could have a significant impact on reducing the risk of mental health problems among young victims of bullying.

Another possibility refers to the fact that poor adult health outcomes are a function of the persistence of early symptoms that developed at the time of the bullying exposure. For example, mental health problems like depression and anxiety are likely to persist, especially when they manifest early in life (Costello, Mustillo, Erkanli, Keeler, & Angold, 2003). Furthermore, most adult psychiatric disorders are preceded by a juvenile history of mental health problems: 75% of adults with a diagnosis for a psychiatric disorder had met diagnostic criteria before the age of 18, 50% prior to the age of 15 (Kim-Cohen et al., 2003). Untreated signs of psychological distress that appear early in life could be the precursors to a life of poor health, both mental and physical. Early interventions targeting early symptoms of mental health problems could successfully mitigate poor outcomes among bullied children as these symptoms can become chronic and persist into adulthood.

Although research findings show that being bullied independently contributes to adjustment problems, it does not operate in isolation. Children who are the victims of bullying are not only at risk of developing early symptoms of mental health problems. They enter a cycle of violence and abuse that may perpetuate itself over time and across settings (Finkelhor, Ormrod, & Turner, 2007b, 2007c). Therefore, being bullied in childhood is often preceded by other forms of abuse at home, and followed by further abuse from peers or adults, forming the first stages in a cycle of victimisation that perpetuates over time and across situations. Although empirical evidence indicates that each different form of abuse independently contributes to poor outcomes, it may be the accumulation of various types of violence exposure in childhood that is at the source of physical and mental health problems in later life, more so than only one type alone (Finkelhor et al., 2007a, 2007b).

Psychological mechanisms including emotional and social-cognitive processing have also been associated with peer victimisation and bullying and could account for the persistence of its associated poor outcomes. For example, appraisals of control (Catterson & Hunter, 2010), hostile attributions and social perspective awareness (Hoglund & Leadbeater, 2007) and coping self-efficacy (Singh & Bussey, 2010) have all been associated with peer victimisation, and mediation analyses further revealed that they accounted for various measures of adjustment problems such as loneliness, social anxiety and withdrawal during adolescence (Catterson & Hunter, 2010; Hoglund & Leadbeater, 2007; Singh & Bussey, 2010). Furthermore, poorer emotion recognition abilities have been observed among victims of relational bullying, and especially for emotions of anger and fear (Woods, Wolke, Nowicki, & Hall, 2009). These findings suggest that interventions aimed at changing such cognitive appraisals could be helpful in preventing the development, and perhaps also the persistence, of mental health problems among victims of bullying.

Being bullied in childhood has a pervasive impact on victims’ lives. Another process through which bullying may impact later outcomes refers to the damaging effect of childhood bullying victimisation on several domains and not only one aspect of individuals’ development. Indeed, being bullied in childhood has been shown to have a detrimental effect on life opportunities for building the human and social capital young children need to overcome adversity and live successful and fulfilling lives. The studies reviewed above show that bullied children end up lacking social relationships, having poor physical health and suffering from financial difficulties as adults. These findings indicate that a lack of resources and support may be a plausible pathway to explain the persistence of poor health outcomes among young victims of bullying.

Although described separately, these processes are likely to operate together in contributing to atypical development. Multidisciplinary research across different levels, from biological embedding of stress to poly-victimisation, is essential to understand the underpinning of mental health difficulties among victims of bullying. Animal models may also provide useful insight here because they allow for direct manipulation of bullying exposure (or social defeat) and offer an opportunity to explore biological mechanisms in more depth. For example, an experiment on mice demonstrated the role of brain-derived neurotropic factor (*BDNF*) in the mesolimbic dopamine pathway to explain social aversion among mice exposed to repeated aggression (Berton et al., 2006). Additional studies like this one will guide and orient future human research aimed at understanding the development of mental health difficulties in young victims of bullying.

## Antibullying policy

Considerable efforts are in place to reduce bullying behaviours and limit its impact on the victims. The UK Government’s approach to bullying is summarised in a document which outlines the remit of schools for tackling bullying, their legal obligations, and some effective antibullying strategies (Department for Education, 2017). It provides a definition of bullying, reviews the safeguarding of children and young people and the underpinnings of criminal law. It also provides advices to teachers and school staff on how to tackle and prevent bullying. Attention is also given on how to attend to young victims of bullying. Since the late 90s, all schools in the United Kingdom must have in place an antibullying policy. These policies include – among other information – principles and values of the school, a definition of bullying, and advice on how to record and report bullying incidents. This document must be presented to and discussed with the pupils as well as shared with parents and school staff. Each school develops its own policy and framework for tackling bullying with guidance from the Government. All schools have the ownership of their policies, and as a consequence, their content and implementation vary considerably from one school to another. Furthermore, there has not been any evaluation for determining the impact of this national initiative on reducing bullying behaviours and their consequences on youth mental health and well-being.

Australia is one of the first countries to have developed a national policy for the prevention and management of bullying and other aggressive behaviours, the National Safe Schools Framework (NSSF). This framework lists 11 principles to assist schools in providing a safe environment to their pupils. These include: promote care, respect and cooperation and value diversity; recognise the critical importance of preservice and ongoing professional development in creating a safe and supportive school environment; focus on policies that are proactive and oriented towards prevention and intervention; and take action to protect children from all forms of abuse and neglect. Comparisons of cross-sectional data across 4 years indicate that rates of bullying have only moderately declined and reports from staff suggest poor development and implementation of the NSSF strategies (e.g. few received training, limited funds invested in bullying) (Cross et al., 2011).

Findings from the United States are somewhat more encouraging. A recent study examined the effectiveness of the antibullying legislation using data from 25 different states. Students living in a state complying to at least one guideline recommended by the Department of Education had a 24% reduction in reporting of being bullied (Hatzenbuehler et al., 2015). Findings further reported the legal components that were consistently associated with a reduction in bullying victimisation: statement of scope, description of prohibited behaviours, and requirements for districts to develop and implement local policies. In other words, details, specificity and clarity of the legislative components were all associated with greater success.

A study reported on the changes in bullying behaviours, mental health and mental health service use in Finland (Sourander, Lempinen, & Brunstein Klomek, 2016). A compelling feature of this study is that it capitalises on data collected before and after the introduction of a nationwide school-based antibullying programme in 2009 in this country. Findings indicated no decrease in rates of bullying behaviour between 2005 and 2013, despite the implementation of antibullying programmes nationwide. The authors also noticed no increase in mental health problems between 1989 and 2003, but an increase in mental health service use during that same period. The authors suggest that a combination of antibullying and mental health interventions may offer better results. This is an interesting conclusion that deserves further attention.

## Antibullying interventions in schools

Numerous school-based prevention and intervention programmes have emerged in recent years with the aim of reducing bullying behaviours. Such programmes vary widely with regard to their focus and methods of delivery. For example, some interventions target the implementation of new curriculum. They commonly include videotapes, lectures and discussions around the topic of bullying with the aim of promoting attitudes against bullying and prosocial behaviours. They are usually limited in time and in outreach by involving mostly classrooms for a few weeks. Instead, a whole-school approach implements rules and sanctions school wide, trains teachers in methods for handling bullying, teaches conflict resolution strategies and offers counselling support. It also involves a wide range of people including all pupils, teachers, school staff, families and when possible, communities. Examples of such programmes are the well-known Olweus Bullying Prevention Program (Olweus, 1994) and the KiVa Anti-Bullying Program (Salmivalli, Kaukiainen, & Voeten, 2005). The KiVa programme, a whole-school intervention based on social-cognitive theory, is one of the most widely used interventions and one that combines several elements offered by other programmes.

KiVa was built from two lines of research, one on aggressive and bullying behaviours and one on the participant roles of bullying (Kärnä et al., 2011). This intervention programme includes a combination of universal and indicated actions to prevent and stop the occurrences of bullying incidents. The universal actions focus at influencing youth’s reaction when witnessing bullying instances (bystanders). The idea here is to change the attitude of the classmates in order to reduce the reward and the motivation of those who bully others. The emphasis is on empathy, self-efficacy and antibullying attitudes. The indicated actions focus on the victims and the bullies more specifically. This programme is not limited to implementing a school ethos and goes beyond by providing staff practical tools such as video films, computer games, and Internet forum. This programme has been shown to be effective at reducing all forms of bullying, including exclusion, cyber and threats, between 21% up to 63% in older pupils (Salmivalli, Kärnä, & Poskiparta, 2011) and also with younger pupils, both self- and peer-reported (Kärnä et al., 2011).

Systematic reviews have evaluated the effectiveness of antibullying programmes more generally and provide encouraging findings with slightly greater reduction in bullying behaviours than bullying victimisation and associated poor outcomes (Ttofi & Farrington, 2009a, 2011; Vreeman & Carroll, 2007). Overall, school-based antibullying programmes reduced victimisation on average by 17%–20% (Ttofi & Farrington, 2011). Greater reduction in victimisation was found for intensive and holistic approaches involving multiple groups of people and environments. Factors associated with better results included parent training, improved playground supervision, disciplinary methods, school conferences, videos, information for parents, work with peers, classroom rules and management (Ttofi & Farrington, 2009b). Efficient antibullying programmes are important and should be developed and supported as widely as possible. However, these programmes are likely to be costly and challenging for schools from deprived areas which deal with several other important educational challenges. Furthermore, evaluations of antibullying policies and school programmes tend to suggest that the likelihood of eradicating bullying behaviour is small and despite such invaluable programmes, a considerable proportion of young people will not escape this form of abuse in their youth. While rigorous study designs and methodology are needed to advance the examination of the efficiency of these important programmes (Bradshaw, 2015), efforts and funds should also be invested in interventions focused on limiting distress and adjustment difficulties among young victims and possibly by the same token, preventing long-lasting problems in later life.

## Involving potential victims in prevention programmes

It might be considered controversial to investigate early factors that could increase the risk of children and adolescents becoming victims of bullying. This endeavour goes against a general assumption that bullying has nothing to do with the unfortunate victims, but all to do with the perpetrators of bullying behaviours. However, the search for these predictors is central to our understanding of the impact of being bullied in childhood. It is crucial for research to account for these factors when determining later outcomes associated with being bullied in childhood. From a prevention perspective, it is also imperative to identify characteristics that render children vulnerable for bullying victimisation (Espelage, 2016).

Although prospective longitudinal studies remain the exception in this line of research, findings indicate that both contextual and individual factors are associated with youths’ risk of being bullied. A meta-analytic investigation and empirical studies have reported that being the victim of bullying, including being a bully/victim, is associated with a range of factors including male gender, young age, low social competence, difficulties solving social problems and social rejection/isolation (Analitis et al., 2009; Bowes et al., 2009; Cook, Williams, Guerra, Kim, & Sadek, 2010). In line with the definition of bullying, research has demonstrated that victims of bullying are a vulnerable group who show difficulties prior to being bullied. Some longitudinal studies report an increased risk of being bullied in childhood associated with early emotional problems, such as withdrawal, anxiety or depression (Arseneault et al., 2006; Bond, Carlin, Thomas, Rubin, & Patton, 2001; Kaltiala-Heino, Fröjd, & Marttunen, 2010; Lester, Dooley, Cross, & Shaw, 2012; Siegel, La Greca, & Harrison, 2009). In addition, preschoolers who display aggressive behaviours (Barker, Boivin et al., 2008; Jansen, Veenstra, Ormel, Verhulst, & Reijneveld, 2011; Snyder et al., 2003) and attention-deficit/hyperactivity and oppositional defiant problems (Verlinden et al., 2015) are more likely to experience peer victimisation and bullying in the school years.

The role of families has also been emphasised as an important factor associated with the risk of being bullied (Beran & Violato, 2004; Jansen et al., 2011; Lereya, Samara et al., 2013; Wolke & Skew, 2012b): low parental educational level, negative parenting such abuse and neglect, poor communication, material deprivation, parental depression, lack of supervision and involvement, and low socioeconomic status have all been associated with small to moderate risks of being a victim of bullying and being a bully/victim. Other contextual factors associated with bullying victimisation include school characteristics such as overcrowding and the number of children receiving free school meals (Barnes, Belsky, Broomfield, & Melhuish, 2006; Bowes et al., 2009).

Twins studies have pushed further the search for factors associated with being bullied by showing it is partly heritable. One study found that genetic influences accounted for over two-thirds of individual differences in children’s bullying victimisation during the first 2 years of their formal schooling (Ball et al., 2008). This finding does not imply there is a gene for being bullied in childhood. Rather, it suggests that heritable symptoms such as emotional and behavioural problems mediate these genetic influences. Environmental factors not shared by people in a family accounted for the remaining variance in bullying victimisation, supporting a study which has shown that the environment also influences children’s risk of peer victimisation (Brendgen et al., 2008).

The mechanisms explaining how specific characteristics and environments translate into a risk for children being bullied are not fully understood: anxious and depressed children may be perceived as easy targets who will not retaliate when other children are abusive towards them. Aggressive children may attract hostility from other children. Contextual factors may also influence child characteristics, which in turn affect their risks for being bullied. For example, one study has shown that individual characteristics including aggressiveness, social isolation, academic performance, prosocial behaviour and dislikability accounted for the effect of social circumstances on preadolescents’ risks for being bullied (Veenstra et al., 2005). However, another study indicated that despite control for children’s emotional and behavioural problems, physical maltreatment and school overcrowding were independently associated with being bullied (Bowes et al., 2009). Thus, factors in children’s family and school environments may increase their likelihood of being bullied, over and above their personal characteristics.

There is no such thing as a profile for the typical young victim of bullying. In addition to contextual and individual factors, circumstances such as moving to a new school or starting to wear glasses may also put some children at risk of being bullied. However, evidence indicates that youths from deprived socioeconomic backgrounds, who have previously experienced violence victimisation and who already show a vulnerability for developing mental health problems have an increased risk of being bullied, via both genetic and environmental pathways. This body of research has identified individual and contextual factors among children and adolescents that contribute at making them potential victims of bullying. It is important for prevention strategies to consider these factors because they could become targets of fruitful early interventions to stop some children from being bullied in the first place.

A public health approach aimed at preventing vulnerable children from becoming the targets of bullying may be an effective strategy to reduce society’s burden related to bullying. For example, instructing young children (and especially those at risk of becoming the target of bullying) skills for facing adversity and standing up to bullying may contribute to reducing this form of abuse. Prevention programmes aimed at building resilience could also benefit young children likely to be exposed to this form of abuse. Providing children with tips on how to make and keep friends may be an example of such intervention (van Harmelen et al., 2017) and this may be especially important in this era of digital age when children and adolescents are spending more time on mobile devices. Involving families could also be an additional asset of such programmes (Bowes et al., 2010). However, it is important to remember at this point that young children who are victims of bullying already show signs of vulnerability and are possibly at risk for developing difficulties despite their experience with bullying. While prevention and intervention programmes may improve the lives of young victims by reducing the likelihood of one form of abuse, it is unlikely that alone, they will solve all youths’ problems.

## What next?

The evidence reviewed above provides strong and robust support for an independent contribution of childhood bullying victimisation to the development of poor outcomes throughout the life span, including mental, physical and socioeconomic outcomes. However, several important questions remain unanswered. Here are a few.

First, there are increasing concerns about the impact of cyberbullying and Internet harassment. This form of abuse deserves careful attention given the widespread use of social media by young people today. While it is not clear whether harassment on the Internet and social media is a true form of bullying (the perpetrator being sometimes anonymous, it may not always be a form of peer victimisation where power imbalance exist), it has been associated with symptoms of mental health problems (for a review see George & Odgers, 2015) and has even been found to be more strongly associated with suicide ideation compared to traditional forms of bullying by some (van Geel, Vedder, & Tanilon, 2014) but not others (Przybylski & Bowes, 2017). The anonymity conferred by online interactions may further empower the perpetrators because they know they are less likely to face the consequences of their actions. Cyberbullying remains, however, a less frequent form of harassment compared to other types of bullying (Olweus, 2013; Przybylski & Bowes, 2017; Smith et al., 2008) and needs to be examined in the context of other forms of victimisation to ensure its independent contribution to poor outcomes.

Second, considerable attention has been focused on bullying in the childhood and adolescent years. Bullying also takes place among adults with potentially damaging consequences, domestic violence potentially being one such example. Some research has been conducted among specific groups such as prisoners (Ireland, 2011) but this line of work could be extended to representative population of adults. For example, bullying in the workplace has gained considerable interest recently. Institutional bullying operates within an organisation’s rules and policies and takes place, typically but not exclusively, during the adult years. There are suggestions that this form of bullying affect workers’ morale and productivity. Research should determine whether it also contributes to mental health problems among adults, as this would also have an important economic impact.

Third, the role of genetic factors has been neglected when it comes to understanding the impact of being bullied in childhood. It is important to consider genetic influences to fully recognise the extent to which bullying affects poor outcomes in later years and identify most at-risk groups. It is also important to explore the genetic influences that contribute to the risk of being bullied. This may provide fruitful avenues for preventing young children from being bullied in the first place. As an example, the use of polygenic risk scores could help identify heritable characteristics associated with the risk of being bullied at a young age.

Fourth, the examination of the outcomes associated with childhood bullying victimisation should not be limited to individual consequences and could be extended to societal impacts, including institutions and systems. Emerging studies on the mental health service use are good examples. Research could include measures of the consequences of bullying victimisation on health institutions, social services and the education system. In addition, studies could also include measures of economic impact.

Fifth, developing new innovative and rigorous research designs remains crucial despite the strong evidence reviewed above showing that being bullied in childhood can have a significant harmful impact. The use of natural experiments and other innovative study designs to support causal inferences of the role of bullying victimisation could strengthen current evidence. The use of animal models, where researchers can exercise greater control over the environment, can help unravel the mechanisms behind poor outcomes associated with being bullied. Modifications in animal social hierarchies are well suited to examine the impact of bullying victimisation and easily allow the observation of associations between changes in social status and changes in outcomes. Natural experiments such as the discordant monozygotic twin design also have the potential to strengthen conclusions by controlling for a wide range of confounding factors including genetic influences. Better control of confounding variables and especially other forms of victimisation is also crucial. The use of propensity score models (Jaffee et al., 2012) could help strengthening the evidence accumulated thus far.

Sixth, there is a lack of neuroimaging findings on structural and functional brain differences among children and adolescents’ victims of bullying. Based on recent review of studies in youths who experienced maltreatment (McCrory, De Brito, & Viding, 2010), we would expect an effect of bullying on some brain structures and/or functioning. Seventh, intervention programmes should be systematically evaluated to inform on the effectiveness of what we are currently doing to stop bullying, what works and what we need to change.

## Conclusions

Based on existing evidence thus far, bullying should be considered as another form of childhood abuse alongside physical maltreatment and neglect. Several rigorous studies reviewed above provide strong and robust support for an independent contribution of childhood bullying victimisation to the development of poor outcomes throughout the life span, including mental, physical and socioeconomic outcomes. Further research is needed to better understand the mechanisms explaining the emergence and the persistence of these poor outcomes. In the meantime, efforts focusing on stopping bullying behaviours should not only be supported but also be widened to provide appropriate help to the young victims and prevent children and adolescents from becoming the target of bullying.

## Key points

- Research has shown that being bullied in childhood contributes to children’s and adolescents’ adjustment problems and can lead to poor outcomes throughout the life span, including mental, physical and socioeconomic difficulties.
- Efforts aimed at decreasing bullying behaviour should reduce associated problems among young victims.
- Current antibullying programmes have provided encouraging findings; however, it is unlikely they will eradicate bullying behaviours. This leaves youths vulnerable to becoming targets of bullying behaviours, and to experiencing difficulties associated with having been bullied.
- To reduce poor outcomes associated with childhood bullying victimisation, interventions could widen their scope to focus on increasing resilience among young victims of bullying and on reducing the risk of victimisation among vulnerable youth.
